# Race, Breastfeeding Support, and the U.S. Infant Formula Shortage: An Exploratory Cross-Sectional Study

**DOI:** 10.3390/healthcare14020148

**Published:** 2026-01-07

**Authors:** John P. Bartkowski, Katherine Klee, Stephen Bartkowski, Ginny Garcia-Alexander, Jacinda B. Roach, Shakeizia (Kezi) Jones

**Affiliations:** 1 Department of Sociology and Demography, University of Texas at San Antonio, San Antonio, TX 78249, USA; 2 Bartkowski & Associates Research Team, San Antonio, TX 78258, USA; katie@bartconsulting.org (K.K.); stephen@bartconsulting.org (S.B.); 3 FLO Analytics, 3140 NE Broadway Street, Portland, OR 97232, USA; ggarcia@flo-analytics.com; 4 Mississippi Public Health Institute, 5 Olympic Way, Madison, MS 39110, USA; jroach@msphi.org (J.B.R.); kjones@msphi.org (S.J.)

**Keywords:** lactation, breastfeeding, race, ethnicity, African American, child, infant, mother, United States, South, Mississippi, rural

## Abstract

**Background/Objectives:** African American women are less likely to breastfeed in general and to breastfeed exclusively for the first six months of infancy. Racial and ethnic breastfeeding disparities are especially pronounced in the South, particularly in rural communities. These differences are attributed largely to structural lactation impediments that include less breastfeeding support in healthcare settings, workplaces, and communities. While a great deal of research has explored racial differences in breastfeeding, minimal attention has been paid to the social correlates and racial disparities associated with the 2022 U.S. infant formula shortage. Our study explores racial distinctions in the formula shortage’s effect on breastfeeding support among Gulf Coast Mississippians. **Methods:** We use data from the second wave of the Mississippi REACH (Racial and Ethnic Approaches to Community Health) Social Climate Survey to determine if racial differences are evident in the formula shortage’s influence on breastfeeding support. We predict that the infant formula shortage will have prompted African American respondents to become much more supportive of breastfeeding than their White counterparts, net of sociodemographic controls. This hypothesis is based on the lower prevalence of exclusive breastfeeding among African Americans, thereby indicating a greater reliance on formula. The study uses a general population (random digit dial) sample and purposive (exclusively African American) oversample to analyze validated data from a cross-sectional survey. Sampling took place between September and December 2023, with a sample population of adult male and female Mississippians. A series of binary logistic regression models were employed to measure the association of race with breastfeeding support changes resulting from the infant formula shortage. **Results:** The study results support the hypothesis, as seen by a positive association between African Americans and increased breastfeeding support directly related to the infant formula shortage. Further, the baseline statistical model reveals African American respondents to be five times more likely than White respondents (*p* < 0.001) to report that the formula shortage increased their support of breastfeeding. **Conclusions:** We conclude by discussing this study’s implications and promising directions for future research.

## 1. Introduction

In 2025, the Mississippi State Department of Health declared a public health emergency in response to rising infant mortality rates across the state. Data from 2024 highlights the infant mortality rate increase (8.9 to 9.7 infant deaths per 1000 live births) across Mississippi, with 3527 infant deaths since 2014 [1]. Mississippi also leads the United States in racial and ethnic mortality and health issues [2,3,4]. Mississippi has a 15% preterm birth rate, 18% for African American Mississippi infants, which is higher than the national average of 10.4% [5]. Mississippi’s infant mortality rate is 9.7, which is greater than the national average of 5.6 [5]. Breastfeeding is a primary protective factor against disease and illness that disproportionately affect African American Mississippians and is championed by the World Health Organization and the American Academy of Pediatrics [6,7,8,9,10]. In 2022, 8.6% of live births in the U.S. indicated low birth weight, with 86 infants with low birth weights born per week in Mississippi [11]. Additionally, despite robust evidence of its overall benefits, breastfeeding rates among African American women are ranked among the lowest of all non-White racial populations throughout the country [12,13,14,15,16]. Evidence suggests that social barriers persist in hindering breastfeeding initiation and duration (i.e., historical and traditional stigma, household accommodations, no or unknown community support, pain, time limitations, unaddressed questions or issues in latching and milk production, unavailability of healthcare professionals) [12,13,14,15,16,17]. Research identifies the remaining effects of generational trauma through slavery wet nurse practices on modern breastfeeding decision-making, especially in the Deep South of the United States [17,18]. Intersectional cultural barriers combine with these historical factors to produce significant impediments that prevent African American mothers and infants from receiving myriad breastmilk health benefits. Therefore, African American women, especially of lower socioeconomic status, have a diminished likelihood of setting breastfeeding goals or initiating breastfeeding. According to the CDC, only 6% of Mississippi hospitals limit infant formula promotion for breastfeeding newborns [19]. Infant formula is defined as a nutrition source “for infants as an alternative to human milk” [20]. Alternatively, breastfeeding is defined as “the process of feeding a mother’s breast milk to her infant, either directly from the breast or by expressing (pumping out) the milk from the breast” and providing it by bottle-feeding the infant [21]. Altogether, lack of social support for breastfeeding and readily promoted infant formula can, in many instances, increase dependence on baby formula [22].

The coronavirus (COVID-19) pandemic produced several adaptations that were maintained or re-occurred during the infant formula shortage of 2022. One study identified that mothers receiving telehealth services during the coronavirus pandemic found that virtual lactation support was moderately effective when compared to in-person support. Virtual support barriers included technical difficulties, challenges assisting with physical breastfeeding issues (i.e., latching), and accuracy in assessing infant growth. However, these barriers were tempered by strengths such as flexibility and convenience, increased communication strategies, and safety from spreading illnesses. Researchers also found that lactation professional visits decreased during the height of the pandemic, with limited in-hospital and pediatrician availability [23]. Another study reported that one third of families who relied on infant formula before the pandemic resorted to less-than-desirable feeding practices during the pandemic due to formula shortages and financial strain. This study result coincides with the 2022–2023 CDC’s findings that more than 30% of children under the age of 5 lived in households that could not always afford nutritious foods [24]. Alternatively, there was also a notable increase in families relying on human milk during the pandemic (46% of families with infants) [25]. Additionally, 97% of participants in another study reported new intentions to exclusively breastfeed their infants in the first six months, with 31% indicating increased frequency of breastfeeding and delayed plans to wean infants due to stay-at-home orders [26].

Following the COVID-19 pandemic, the 2022 United States disruption of the infant formula supply chain caused by contaminated products and closed manufacturing facilities led to an increase of more than 70% in out-of-stock rates throughout the U.S. and several global markets while inducing widespread panic [27]. A monopolized formula milk industry, whereby four corporations control 90% of U.S. infant formula, combined with dependence on limited formula providers per state emphasized the need for exploring alternative infant feeding practices [28]. The impact of the 2022 U.S. infant formula shortage was acutely felt by racial and ethnic groups, along with low-income communities already at a maternal-infant health disadvantage across Mississippi and other states [29]. The Women and Infant Children (WIC) program provides cost-effective formula to infant parents. However, to ensure a low-cost product, WIC uses a competitive bidding process with formula manufacturers to earn the sole WIC formula contract per state, which then provides rebates on purchases. These rebates allow for WIC’s overall cost to be reduced and the program to aid more families. Manufacturers who already monopolize the infant formula market effectively expanded their monopoly in Mississippi among WIC participants. Since the primary WIC benefits are limited to a single brand of products, the brand’s factory closure left WIC recipients especially vulnerable [30,31].

One study explored how the infant formula shortage directly influenced breastfeeding intentions among expectant mothers. Related stress encouraged prenatal feeding goals to encompass greater breastfeeding than before the shortage event [32]. In the midst of the formula shortage, average breastfeeding initiation rates increased by 1.96% across the country, which resulted in historically elevated rates at the conclusion of this crisis event [32]. These increases were most notable among African American mothers, mothers with lower education, participants in the Special Supplemental Nutrition Program for Women, Infants, and Children (WIC), rural county residents, and Medicaid recipients [33]. Additional research identified overall increases in breastfeeding rates after the shortage had concluded in comparison with before the shortage event, where initiation in some areas increased by 10.6% [34]. In addition to limited baby formula availability, more reports were circulated on illness associated with the consumption of infant formula that heightened interest in breastfeeding pursuits. One study examined the use of human breast milk through regulated donors, which are limited in number across the United States. Donor milk banks reported a significant increase in use during the shortage (2% to 28%). Additionally, the use of human milk through informal sharing showed a notable increase (5% to 26%) from pre to post-shortage [35]. All of these findings suggest increased interest in lactation and elevated demand for human milk over formula. 

Alternatively, parents’ feeding decisions for previous children can play a significant role in feeding decisions for new infants, more so, in certain circumstances, than formula or breastfeeding education availability [36]. Therefore, community support (i.e., family support) may have a varying impact on mothers’ infant feeding practices depending on previous formula use versus breastfeeding for their older children. Alongside seeking alternative infant food sources, parents sought support through social media connections, relatives and friend groups, and healthcare providers, which often resulted in moderate-to-low helpfulness. Parents identified a need for a wider array of infant formula availability, free prenatal breastfeeding education, and postpartum breastfeeding support [37]. Mothers also noted increased adverse impacts on their mental health, in part from significant financial costs, which influenced changes in infant feeding practices. However, social and family networks often led to breastfeeding or formula resources and other tangible and intangible supports [38].

The formula shortage event in the U.S. also highlighted racial and ethnic gaps in healthcare and food security, especially among non-White households with infant children. Among families directly impacted by the shortage in infant formula, African American parents had an increased likelihood of reporting disruptive coping (i.e., unintentional heightening of the infant’s stress, discomfort, mealtime negativity) than White parents [39]. In-depth interviews with Hispanic mothers further revealed three primary challenges of the formula shortage: a determination to breastfeed despite a lack of professional help or guidance, efforts to counter shame and stigma if breastfeeding was not an option, and a lack of baby formula availability [40].

Given the generally lower prevalence of breastfeeding among African Americans [12,13,14,15,16,19], the infant formula shortage should have produced greater vulnerability in formula reliance for African American respondents. Therefore, we hypothesized that the infant formula shortage would bolster support for breastfeeding more greatly among African American respondents than among their White peers. This hypothesis is justified by nutritional scarcity theory [41]. Nutritional scarcity theory posits that the limited availability of a particular food will influence consumption appraisals concerning that food. In this case, it is reasonable to predict that breastfeeding will be considered a more attractive alternative to baby formula in the wake of the formula shortage. Because formula becomes an unreliable food source during a shortage, breastfeeding will be elevated in importance as an alternative means of supporting infant nutrition. The effects of scarcity will be more profoundly influential among African American respondents.

## 2. Materials and Methods

The present study analyzes validated data from a cross-sectional survey. This survey was designed to capture the reported prevalence of breastfeeding versus infant formula use in primary social networks, racial identity, education status, and household income, including additional factors. Although this design cannot establish causal relationships, the cross-sectional survey does allow for the establishment of associations between the independent and dependent variables net of controls (confounding factors). The study was conducted as part of the Mississippi REACH Social Climate Survey—Wave II (part of a larger study under the Mississippi REACH program), a follow-up assessment of the community-based project between 2018 and 2023 along coastal Mississippi. Only the second wave of data is used for this study because it is this wave alone that inquired about breastfeeding support changes in relation to the infant formula shortage. (Wave 1 preceded the shortage.) This project (MS REACH) addresses key areas of health disparities disproportionately impacting African Americans across three Mississippi Gulf Coast counties. This study’s population sample was gathered under the counties served by MS REACH. The goal of this survey was to form a general comprehension of African American and White social disparities related to breastfeeding along the Mississippi coast and in areas of similar population and health disparities. An existing memorandum of understanding between the Declaration of Helsinki and the Institutional Review Board of Mississippi State University (IRB-17-04-MOU) provided data collection approval for the general population survey. Data collection for this second wave of the Social Climate Survey was fielded from July through September 2019. This study’s analytical sample was retrieved from a subsample of the full Mississippi REACH Social Climate Survey—Wave II dataset.

### 2.1. Study Sample

The sample used in this study consists of 171 African American respondents and 215 White respondents, from a general population sample combined with a purposive oversample. Oversampling for an additional 118 African American residents was used to more fully capture African American perspectives on health risks and maternal health disparities in the coastal region and nationwide. Therefore, only non-Hispanic African American and non-Hispanic White participant results were analyzed for this study. Why include men as well as women in a survey on breastfeeding support, knowledge, and network prevalence? First, the survey does not inquire about personal breastfeeding practices. Therefore, the survey items are relevant for both men and women. Second, much current work, including the researchers’ own prior publications, views the initiation and duration of breastfeeding as influenced by gender-diverse networks of social support [42,43]. Those networks include men, particularly as family members (husbands, fathers, grandfathers), as well as close friends.

This study utilized two different methods for optimally capturing the intended populations. First, a random digit dialing method for Mississippi cellular (cell) phone numbers to establish a representative sample of adults (18 and older). Sample adult cell phone numbers were drawn from the Mississippi Gulf Coast counties served under MS REACH (Hancock, Harrison, and Jackson). A total of 39,000 relevant population cellular and landline phone numbers were selected, with a maximum of eight dialing attempts per phone number before retirement. The general population sample was supplemented by an oversample of African American adult males and females (18 and older). This oversample was collected through SurveyMonkey using a purposive sampling technique to broadly capture oversample perspectives while maintaining complete parity across the general population sample and oversample surveys. Oversample survey collection was disseminated through MS REACH partner organizations (community centers, congregations, etc.) with outreach conducted by an in-person community team member along the Gulf Coast. A desire to not burden community partners with difficult data collection tracking procedures leaves the research team unable to render a precise completion rate for the oversample; however, due to our collaboration with trusted African American health advocates and organizational partners in the community, we estimate a relatively smaller proportion of refusals than is commonly encountered. Incentives of USD 10 Amazon e-gift cards for each oversample survey completed, with an additional raffle (random drawing) for three USD 100 Amazon e-gift cards. All incentives were provided to qualifying oversample participants. The survey was briefly linked to a website by a partner organization and received 1301 bot completions that were subsequently identified and removed prior to data analyses. Safeguards were put in place immediately following the bot completions to ensure that only Mississippi Gulf Coast residents completed the survey and obtained the incentives. A brief follow-up survey was administered to the same African American respondents who completed the oversample survey to capture a more in-depth perspective on the relationship between the selected independent and dependent variables. This study uses de-identified data from this random sample, selecting participant data from the African American and White racial categories. Inclusion criteria for study participation spanned age (18 or older), race/ethnicity (all races/ethnicities for the primary survey, non-Hispanic African Americans for the oversample), gender (male/female/other), and location (Mississippi REACH counties). Exclusion criteria for potential respondents were younger than 18 years of age and/or not a resident of the three coastal counties within the study parameters. All persons excluded on these bases were informed of their ineligibility to complete the survey and discontinued from participation. Qualifying participants were informed that they could skip any item response or choose to withdraw from the survey at any point. All participants were informed of the voluntary nature of the survey and the publication potential of their de-identified responses. All qualifying 386 adults completed the survey with a cooperation rate of 29.0% for the general adult population survey.

### 2.2. Variables

The key dependent variable for this study is the effect of the infant formula shortage on breastfeeding support among respondents. This construct is measured by the survey item, “How has the 2022 formula shortage affected your support of breastfeeding?” The response options are featured on a five-point scale that includes “Much more supportive,” “Somewhat more supportive,” “No change,” “Somewhat less supportive,” and “Much less supportive.” We recoded this variable into a binary measure in which the new category of “No change or less supportive” (coded 0) consists of original response options 3–5. We then created a comparison “More supportive” category (coded 1), which includes original response options 1–2. This study’s dependent variable is a novel survey item that is warranted for use in an exploratory investigation such as this one. It was pretested and approved for comprehensibility (essentially, face validity) by subject-matter experts and individuals very familiar with members of the surveyed population. The primary independent variable is race because the data are analyzed for different perceptions expressed by African American and White respondents on the Mississippi Gulf Coast. Therefore, the descriptive statistics and odds ratios featured in the tables are all predicated on African American/White comparisons. Other racial groups were eliminated from the analyses due to extremely small case numbers. Comparative data on African Americans in our general population sample (n = 53) and those in our oversample (n = 118) are available by request. We conducted comparisons of these groups on the dependent variable. Response patterns to changes in breastfeeding support due to the formula shortage were quite similar across the general sample of African Americans and their oversample counterparts. Therefore, our use of the combined general population sample and purposive oversample is warranted.

We control for a range of sociodemographic factors that have been included in previous studies of breastfeeding outcomes using this data source and others like it, including education (1 = “Never attended or K only” through 9 = “Beyond master’s degree”), income (1 = “Less than $10,000” through 11 = “$200,000 or more”), age (continuous), gender (Male is reference), employment status (Employed is reference), marital status (Married is reference), and relevant household characteristics (see Table 1) [42,43].We control for lactation network prevalence, operationalized by the following item: “How common is breastfeeding infants among your female friends and relatives?” Response categories include “None of them have breastfed,” “Very few of them have breastfed,” “About one-quarter of them have breastfed,” “About half of them have breastfed,” “A majority of them have breastfed,” and “All of them have breastfed.” This variable is treated as a continuous control variable in this study. We included a breastfeeding knowledge control variable, “To the best of your knowledge, what is the recommended number of months to feed a baby with nothing other than breast milk?” with a write-in response option. We were less concerned with correct or incorrect responses to this item (six months qualifies as correct) but were curious if this measure would exhibit a significant association with the dependent variable. While it did not have a significant association, this item was retained in the full model (see Model 2 of Table 2). Given the importance of religion among African Americans in the South [44], we also control for religious attendance as a continuous variable with the question, “How often do you attend religious services?” Responses to this item range on a nine-point scale from “Never” (=1) to “Several times per week” (=9). 

### 2.3. Analytic Strategies

The survey data were weighted to represent adults 18 years of age and older residing in the Gulf Coast region of Mississippi (Jackson, Hancock, and Harrison counties) at the time of the survey (2023). The data were also weighted to adjust for oversampled African American males and females aged 18–50. Missing data were addressed as follows. We used iterative imputation to estimate and replace all missing values in the dependent, independent, and control variables once we verified that they were missing at random. Missing data was within acceptable ranges (e.g., 7.8% missing for the dependent variable, 0% missing for the independent variable). Additional details (counts, percentages) of missing data for covariates are available by request.

As noted, the dependent variable is the infant formula shortage’s influence on breastfeeding support. This measure was utilized as a binary variable in which “No change or less supportive” was contrasted with “More supportive.” The analysis began by generating descriptive statistics for all study variables. Counts and means or percentages were calculated for each variable included in this investigation. These variables are featured in Table 1. Thereafter, correlations among key study variables were calculated, but consistent with current publication standards, this paper does not feature the correlation matrix. Following these analytical steps, a series of binary logistic regression models were estimated to assess the association of race with breastfeeding support changes resulting from the infant formula shortage. In Table 2, Model 1 estimates the zero-order relationship between race and the influence of the formula shortage on breastfeeding support. This zero-order model features no control variables. Model 2 integrates all controls to discern the impact of these covariates on the relationship between race and the impact of the infant formula shortage on breastfeeding support. These controls are entered into Model 2 to determine the degree to which they attenuate the relationship between the independent variable (race) and dependent variable (breastfeeding support change due to infant formula shortage), whose zero-order relationship is featured in Model 1. A comparison of these models can reveal if the variable relationship established in Model 1 is changed through the addition of variables that previous research has revealed can serve as confounding factors [42,43]. As part of our statistical analyses, we tested for a wide range of possible interaction terms, including race x gender. No interaction terms were statistically significant. Therefore, interaction term results were not included in any models but are available from the lead author upon request. All statistical analyses were conducted using R version 4.3.2.

## 3. Results

Table 1 presents the descriptive statistics associated with the study variables. The dependent variable, breastfeeding support change related to the infant formula shortage, reveals an inverse pattern, respectively, for African American and White respondents. Whereas a robust majority of African American respondents (n = 128) became more supportive of breastfeeding after the formula shortage, only a minority of White respondents (n = 80) did so. (The potential significance of this difference is left to regression models to determine and is addressed in subsequent paragraphs.) Therefore, African Americans overall expressed greater breastfeeding support following the infant formula shortage. White respondents were mostly unchanged or less supportive of breastfeeding following the formula shortage. There are some racial differences in breastfeeding network prevalence, with African American respondents reporting larger proportions of breastfeeding women in their primary social networks. This finding is not altogether surprising because breastfeeding prevalence increased among African American women following the infant formula shortage. An interesting difference emerges concerning knowledge of the recommended number of months for breastfeeding infants. African American respondents wrote in a greater number of months (9.83, on average) than did their White counterparts (7.60). This difference could be attributed, once again, to the differential impact of the infant formula shortage on more favorable perspectives of breastfeeding. We would note that gender proportions of respondents are somewhat different across the racial subgroups featured in this study. African American women were more greatly represented (70.18% of the African American subsample) than their White counterparts (54.42% of the White subsample). A Chi-square test revealed this proportionality difference to be moderately significant (X^2^ = 4.16, *p* < 0.05), thereby underscoring the value of controlling for gender in subsequent regression analyses. All other study variables featured in Table 1 are used as regression model controls in Table 2 and, therefore, do not warrant sustained attention in our Table 1 summary of descriptive statistical patterns.

Table 2 presents the results of two models using logistic regression. Odds ratios are featured in Table 2. Model 1 is a zero-order model that examines the statistical association between the dependent variable, change in breastfeeding support due to the infant formula shortage, and the key independent variable, namely, race, in the absence of statistical controls. Model 2 examines the relationship between the dependent variable and the key independent variable with all statistical controls featured in the model.

The results reported in two binary logistic regression models support the hypothesis that predicted the infant formula shortage’s effect on breastfeeding support would be more pronounced among African American respondents than their White peers. Model 1 of Table 2 indicates a positive and significant association between African Americans and increased breastfeeding support resulting from the infant formula shortage. African Americans are 5.18 times more likely to report that the formula shortage made them more supportive of breastfeeding than their White peers (*p* < 0.001). This zero-order model, therefore, provides robust support for the hypothesis. The full model reveals that, with the addition of control variables, the odds of African Americans expressing greater breastfeeding support due to the formula shortage are somewhat reduced, as signified by the odds ratio of 3.73, but not eliminated. Statistical significance for African American respondents remains pronounced in Model 2 (*p* < 0.01). Both regression models offer strong support for rejecting the null hypothesis and provide robust confidence in the observed differences.

## 4. Discussion

The 2022 infant formula shortage had profound effects across U.S. households. This study uniquely examines the relationship between breastfeeding support by racial groups and limited formula availability in Mississippi during this period. In the United States, Mississippi has among the most pronounced infant and maternal health risks, especially affecting the African American population [2,3,4,5,11]. Breastfeeding is championed as the most nutritious infant feeding option for various health benefits in both mother and infant [6,7,8,9,10]. However, breastfeeding rates, although increasing during pandemic-era stay-at-home orders, remain relatively low among African Americans [12,13,14,15,16]. Nutritional scarcity theory outlines potential social negotiation impacts in food selection when presented with compounding factors, such as economic uncertainty, food item availability, and supply chain limitations [41]. The survey results from this study support the hypothesis that the infant formula shortage had a statistically significant effect among African American respondents, more so than their White counterparts, controlling for religiosity, household income, gender, and other sociodemographic factors (see Table 2). Consistent with nutritional scarcity theory, we note a positive association between African Americans and increased breastfeeding support resulting directly from the infant formula shortage. In the zero-order regression model, African American respondents had five times greater odds than White respondents of reporting that the formula shortage made them more supportive of breastfeeding. There are several social dynamics that may explain this racial difference in formula shortage responses.

Recent studies have identified the impacts of breastfeeding messaging across racial groups [42,43,44]. One study suggests that African American women are more likely to be exposed to breastfeeding education through pediatric care doctors or doulas rather than social media, local communities, or friends and family, but this relationship does not result in significant increases in breastfeeding initiation [42]. In another study, African Americans reported greater exposure to pro-breastfeeding media messaging than Whites, but this messaging did not result in increased breastfeeding participation [43]. However, when community support for breastfeeding (i.e., breastfeeding acceptance in public spaces) is perceived by African American women in higher socioeconomic statuses, they are more likely to be exposed to breastfeeding experiences in their social networks [44]. It is therefore possible that perceived community acceptance is more influential on individual breastfeeding support in African American communities than their White counterparts. This finding corresponds with the results where African American participants reported greater proportions of breastfeeding women in their primary social networks (i.e., friend groups, families, coworkers). This finding is reinforced by the increase in breastfeeding prevalence among African American women following the infant formula shortage [33].

When the formula shortage occurred shortly after the height of pandemic social restrictions, healthcare professional support was scarce [37,40] and pro-breastfeeding media was undermined by a lack of familiarity and education in infant feeding options across the United States [37]. African American individuals may have more readily turned to available social supports, such as friends, families, breastfeeding support groups, religious congregations, and others to receive general support, key information, and guidance on breastfeeding. Research has noted that frequent religious service attendance is positively linked with increased breastfeeding initiation, although breastfeeding duration remains weakly associated [45,46]. Religious community-based maternal and infant health programs are significant sources for underserved populations, as with African Americans in coastal Mississippi. Directly related to community support, social network prevalence, and individual support is the perceived number of recommended months for breastfeeding infants. Healthcare professionals recommend exclusive breastfeeding for the first six months of infancy, then encourage breastfeeding for a full 12 to 24 months for maximum infant nutrition [6,7,8,9,10]. African American participants averaged nearly 10 months for recommendations, whereas White participants averaged nearly 8 months. The African American community response is closest to the minimum ideal of 12 months, which suggests a greater amount of nutrition-related knowledge-sharing or witnessed practices among African Americans. Additionally, exposure to breastfeeding often results in gaining factual knowledge and positive perspectives concerning the practice [43]. The majority of White participants remaining unchanged, or having less support for breastfeeding after the shortage, is supported by previous research that shows White women’s rates of breastfeeding as consistently higher than rates among women of color [12,13,14,15,16]. Therefore, the impact of the formula shortage may have been minute for some White mothers, their families, and their social networks. Additionally, White individuals report lower proportions of breastfeeding women in their primary social groups. This circumstance can result in limited first-hand knowledge and pro-breastfeeding dialogue with trusted sources, such as family and key community members. Further, mothers with prior experience breastfeeding or formula feeding their older children may have established preferences and community ties that encourage the continued use of the familiar infant feeding practice.

### Limitations and Future Research

The current study explored a unique relationship between racial groups, individual breastfeeding support, and social network influences that emerged from the infant formula shortage. However, a few limitations noted here suggest potential steps for future research. The study’s survey prioritized the relationships between influential social factors and breastfeeding support, such as community support, related knowledge, and direct or indirect breastfeeding experience. Yet, a cross-sectional study cannot ascertain causal factors responsible for White respondents’ largely unchanged or diminished support of breastfeeding or increased lactation support among African American respondents. Longitudinal survey research remains an attractive prospect to address such questions. Future study pathways could also expand on our results through qualitative measures that allow for more depth in participant responses. The study’s analysis utilized data from a follow-up community assessment of the Mississippi Gulf Coast related to racial and ethnic breastfeeding disparities. This survey was revised from a baseline community assessment, with items related to the formula shortage newly added for this second wave of data collection. This dependent variable item was not featured on the first wave of the survey, which preceded the formula shortage. As noted, cross-sectional data are used in this study. Therefore, no causal claims can be made. The novel survey items (changed views of breastfeeding support due to the shortage) have an “effects” element built into the question, essentially asking participants to identify how their perceptions changed because of the formula shortage. However, this study does not permit the identification of causal pathways through statistical analysis.

Future research would benefit from conducting at least two waves of data collection to comparatively analyze long-term impacts and sustained patterns related to infant formula and breastfeeding education access. Additionally, exploring alternative correlations between attitudes and behaviors related to breastfeeding and formula availability would enhance future maternal and infant health studies and public health strategies. Multi-wave research could therefore explore the prospect for changes in breastfeeding support, if any, that endure well past the shortage. This exploration is also important because it is possible that lower levels of breastfeeding support among African Americans at the outset of the shortage provided an opportunity for greater gains in breastfeeding support due to the shortage. White respondents’ gains in support could be constrained by a ceiling effect (higher initial support levels) not affecting African Americans. Future research could also help better establish the validity and reliability of the dependent variable, namely, changes in breastfeeding support due to the formula shortage, which was deemed comprehensible (sound face validity) for this investigation’s study population but could benefit from testing with other groups. This study’s population of focus prioritized African American and White population comparisons. Although the general population of the United States is widely varied with larger percentages of minority racial and ethnic groups, Mississippi’s largest populations consist of African American and White individuals in the coastal counties that were surveyed. There are also significant health disparities between these two primary groups. Future studies would contribute to the maternal and infant health field by assessing Hispanic and other minority groups’ relationships between community support and breastfeeding in southern United States locations.

Additionally, this oversample survey was initially included on a partner organization’s website and garnered bot completions. All bot responses were identified and removed, and the survey was ultimately completed through phone calls and in-person canvasing attempts. Online surveys are recommended to have surveyors who conduct one-on-one survey completions to prevent participation from respondents who fall outside of the established study parameters. As this was a second-wave survey, the research team examined the content validity of each survey item specific to Mississippi Gulf Coast communities with extensive input from project partners who lead and reside in these communities. However, future studies examining similar topics would benefit from cross-checking content validity for specific populations. Similar to the baseline survey, the current survey items related to breastfeeding community support were adapted from a CDC survey with confirmed construct validity. Future studies can build upon our research methodology and study results by including item validity tests for specific populations across race/ethnicity, gender/sex, education, religion, and household income.

## 5. Conclusions

The 2022 infant formula shortage impacted worldwide infant feeding practices. In the United States, Mississippi’s population was particularly affected. Mississippi has among the most pronounced racial disparities for infant and maternal health in the U.S. Moreover, Mississippi has a large proportion of African American mothers enrolled in WIC (Women and Infant Children), a program that provides breastfeeding assistance and formula procurement services. The availability of breastmilk alternatives, prevalence of breastfeeding media messaging, access to community and healthcare education, and personal experience with breastfeeding combine to exert a powerful influence on individual support for breastfeeding. The majority of White survey respondents indicated no attitudinal change or were less supportive of breastfeeding as a result of the formula shortage. In stark contrast, African Americans reported significant increases in viewing breastfeeding more positively because of the formula shortage. Our use of an oversample of African American Mississippians allowed for a more thorough comparison of African American and White viewpoints on breastfeeding support in the wake of the formula shortage. 

Our study has broader implications for communities exhibiting similar demographic profiles and breastfeeding patterns. The continued use of media messaging and community education efforts across racial groups offers the potential for improved breastfeeding support at the individual level. Introducing policies that counteract breastfeeding stigma and promote community support groups in a variety of settings could positively impact maternal and infant health. Future research would benefit from expanding lines of inquiry into other racial minorities’ experiences with breastfeeding and baby formula promotional efforts. Qualitative research (e.g., focus groups) could shed additional light on media messaging interpretations, social network dynamics, and other influences we were unable to investigate here. Additional knowledge could also be gained by exploring the linkages between healthcare professionals, community efforts to promote specific infant feeding practices, and timelines of breastfeeding or formula use by mothers in areas with varied racial populations.

## Figures and Tables

**Table 1:** Descriptive statistics for all study variables.

Variable	African American		White	
	Count (Mean)	% (SD)	Count (Mean)	% (SD)
Breastfeeding (BF) Support Change from Formula Shortage				
No Change or Less Supportive of BF	43.00	25.15%	135.00	62.79%
More Supportive of BF	128.00	74.85%	80.00	37.21%
Breastfeeding Network Prevalence (mean + SD)	1.76	0.43	1.36	0.48
Recommended Months BF (mean + SD)	9.83	5.34	7.60	3.85
Year Born (Age) (mean + SD)	1971	17.90	1971	16.30
Education	5.61	1.71	5.86	1.73
Religious Attendance, continuous in logit models	6.35	2.46	4.70	2.85
1. Never	12		55	
2. Less Than Once a Year	9		11	
3. About Once or Twice a Year	7		16	
4. Several Times a Year	15		12	
5. About Once a Month	5		31	
6. 2 to 3 Times a Month	18		21	
7. Nearly Every Week	25		11	
8. Every Week	54		42	
9. Several Times a Week	26		16	
Income (mean + SD), continuous in logit models	5.50	2.53	7.00	7.30
**Gender**				
Male	51.00	29.82%	98.00	45.58%
Female	120.00	70.18%	117.00	54.42%
**Adults in Home**				
1 Adult	59.00		50.00	
2 Adults	67.00		114.00	
3 or More Adults	45.00		51.00	
**Child in Home (1 or More Resident Children)**				
0	88.00		136.00	
1 or More	83.00		79.00	
**Employment Status**				
Employed	108.00		132.00	
Not Employed	63.00		83.00	
**Marital Status**				
Married	57.00		126.00	
Not Married	114.00		89.00	
N	171.00		215.00	

Note: Bold and underlined text in the table denotes population subgroups for the study participants.

**Table 2:** Logistic regression models to predict racial differences in breastfeeding support.

Variable	Model 1	CI (95%)	Model 2	CI (95%)
African American (reference: White)	5.18 ***	[0.99–2.34]	3.73 **	[0.56–2.12]
Breastfeeding Network Prevalence			1.06	[−0.16–0.29]
Recommended Months BF (Knowledge)			0.99	[−0.09–0.07]
Year Born (Age)			0.99	[−0.04–0.01]
Education			0.89	[−0.32–0.09]
Religion			1.13	[0.00–0.25]
Income			0.92	[−0.25–0.09]
Gender (reference: Male)			1.2	[−0.49–0.86]
Two Adults in Home (reference: One Adult)			0.72	[−1.18–0.53]
Three+ Adults in Home (reference: One Adult)			0.73	[−1.27–0.61]
Child in Home			1.25	[−0.51–0.96]
Employment Status (reference: Employed)			0.76	[−1.09–0.53]
Marital Status (reference: Married)			1.17	[−0.63–0.94]
Pseudo R-squared	0.047		0.079	
N	386		386	

Note: Breastfeeding support is measured as resulting from the infant formula shortage (weighted). CI represents confidence interval. Actual *p*-values for the primary independent variable (African American) are as follows. Model 1 (*p* = 0.000); Model 2 (*p* = 0.002). Legend: *p* < 0.05 *; *p* < 0.01 **; *p* < 0.001 ***.

## Data Availability

Data are not available due to privacy and ethical restrictions. Because these data are proprietary (i.e., held in a private repository), they are not publicly available or otherwise allowed to be circulated. More information about the data can be provided by the first author upon request.
